# Supplementary material for: Optimal truss design with MOHO: A multi-objective optimization perspective
Source: PLoS One. 2024 Aug 19;19(8):e0308474. doi: 10.1371/journal.pone.0308474 (PMC11332947; doi:10.1371/journal.pone.0308474)
Supplement: Attachment — Submitted filename: Response to Reviewers.docx [file pone.0308474.s001.docx]

**Author's Response to the Review Comments**

Journal: **PLOS ONE**

Ms. Ref. No.:  **PONE-D-24-17050**

Title of Paper: Optimal Truss Design with MOHO: A Multi-Objective Optimization Perspective

Status: Invitation to revise the manuscript

The authors wish to thank the editor-in-chief, co-editors, and reviewers for their insightful comments, positive feedback, and constructive suggestions on the manuscript. These contributions significantly enhanced its quality and readability. We also appreciate the time and effort the editors and reviewers dedicated to the review process.

In response to the feedback, the authors have revised the final manuscript to address all the issues mentioned below. These changes are highlighted in ‘YELLOW’ in the revised manuscript. Detailed responses to the editors' and reviewers' comments are provided. We hope the editors and reviewers find our responses and the subsequent revisions satisfactory.

Reviewer #1: Title of the manuscript: “Optimal Truss Design with MOHO: A Multi-Objective Optimization Perspective”

Summary: The authors propose a new metaheuristic inspired by the movement of hippos to solve multi-objective structural optimization problems from benchmark problems.

1 – The authors use the term “nodal deviation” in the abstract. Could this be more accurately described as “nodal displacement”?

Response: - Thank you for your insightful comment. We agree that "nodal displacement" more accurately describes the concept we are discussing in the abstract and throughout the paper. Nodal displacement refers explicitly to the absolute movement of nodes due to applied loads, which aligns with our analysis and findings. We have revised the manuscript to replace "nodal deviation" with "nodal displacement" for clarity and precision. The changes have been highlighted in yellow in the revised manuscript.

2 - The authors also write size restrictions in the abstract. In optimization, the usual is to write “constraints” instead of “restrictions”.

Response: Thank you for your valuable feedback. We acknowledge that "constraints" are more commonly used and appropriate in optimization. We have revised the manuscript to replace "size restrictions" with "size constraints" in the abstract and throughout the paper to align with standard terminology in the field. These changes have been highlighted in yellow in the revised manuscript.

We appreciate your suggestion, as it enhances the precision and consistency of our terminology.

3 – Please change “segment” to “section” in the paper's outline.

Response: Thank you for your helpful suggestion. We have revised the manuscript to replace "segment" with "section" in the paper's outline and throughout the text where applicable. The changes have been highlighted in yellow in the revised manuscript.

We appreciate your feedback, as it improves the clarity and consistency of our terminology.

4 - Please check that all variable descriptions are correct in Tables 1, 2, and 3. Some variables are written superimposed on each other, for example, in Stages 1, 2, 6, 8, and 14.

Response: Thank you for bringing this to our attention. To ensure accuracy, we have thoroughly reviewed and corrected all variable descriptions in Tables 1, 2, and 3. Additionally, we have resolved the formatting issues that caused variables to appear superimposed on each other, particularly in Stages 1, 2, 6, 8, and 14. The revised tables are included in the updated manuscript; the changes are highlighted in yellow.

We appreciate your detailed feedback, as it helps improve the clarity and presentation of our data.

5 – The authors use the word fitness for the first time in Section 3.2. However, the fitness function has not yet been adequately defined.

**Response**: Thank you for your valuable observation. To address this, we have revised the manuscript to include a clear and comprehensive definition of the fitness function earlier in the paper, specifically Section 3.2. This ensures that readers understand the term correctly before it is used in Section 3.2.

- Fitness is a quantitative value assigned to a solution that indicates its quality or suitability concerning the optimization problem’s objectives.
- Fitness Function: The fitness function is a specific mathematical function that computes the fitness value of a given solution. This function translates the characteristics of the solution into a numerical score based on predefined criteria.

6 – The standard formulation of the optimization problem is incorrect. It is not about maximizing the maximum displacement “ ” but minimising it. Still, using the symbol epsilon to denote displacements is not usual, but rather “u” or “delta”, for example. Epsilon is commonly used to define “deformations”.

Response: Thank you for your insightful comments. The objective is to minimize the maximum displacement, not maximize it. We have corrected the formulation throughout the manuscript to reflect this.

Furthermore, we agree that using the symbol epsilon (ε) to denote displacements is unconventional and may cause confusion. To improve clarity and align with standard practice, we have replaced ε with " δ " to denote displacements. Epsilon (ε) will be reserved for representing deformations.

These changes have been made throughout the manuscript, and all relevant sections have been updated accordingly. The revisions are highlighted in yellow in the revised manuscript.

We appreciate your detailed feedback, as it helps improve the accuracy and clarity of our work.

7 - The authors wrote again: “The primary objective is to maximize the nodal deflection and minimize the total mass…”

Response: Thank you for pointing out this error. We acknowledge that the correct primary objective should be to minimizing the maximum nodal displacement and minimizing the total mass rather than maximizing the nodal deflection. We have corrected this statement throughout the manuscript to reflect the optimisation problem's objectives accurately. These corrections have been made in all relevant sections of the manuscript, and the changes are highlighted in yellow in the revised document.

8 - Is the fitness function the penalty function? The text must clearly define this.

In our study, the fitness and penalty functions serve distinct purposes in the optimization process.

1. **Fitness Function**: The fitness function evaluates the quality or suitability of a candidate solution based on the optimization objectives. It assigns a numerical score (fitness value) to each solution, indicating how well it meets the specified criteria. In evolutionary algorithms, solutions with higher fitness values are preferred and more likely to be selected for reproduction.
2. **Penalty Function**: The penalty function, on the other hand, enforces constraints or bounds on the variables or solutions during optimization. It penalizes solutions that violate constraints by adding a penalty term to the fitness function evaluation. This ensures that only feasible solutions that satisfy all constraints are considered optimal.

To clarify these concepts, we have updated the manuscript to explicitly define and differentiate between the fitness and penalty functions in Section-4, equation -2. These definitions are clearly stated to avoid confusing the two concepts throughout the manuscript. The revisions have been highlighted in yellow for easy identification.

9 – Please change the term “infringement” to “violation”. It is the most common in the context of optimization.

Response: Thank you for your suggestion. We agree that "violation" is commonly used in optimization to describe constraints not satisfied by a solution. We have revised the manuscript to replace instances of "infringement" with "violation" throughout the text, ensuring consistency with standard terminology in the field.

The changes have been implemented across all relevant sections and are highlighted in yellow in the revised manuscript.

We appreciate your feedback, which helps improve the clarity and precision of our presentation.

10 – What values are adopted for the parameters in Equation (2)? The variables C and C_i are used in the same Equation. And these are not adequately defined.

Response: Thank you for bringing up these points. We apologize for the oversight in adequately defining the parameters and variables in Equation (2). Ci signifies the magnitude of violation for each constraint. This penalty function adjusts the objective functions in response to the overall constraint violation, employing penalty factors *ϵ1* and *ϵ2,* the values set to 3 which are determined based on relevant findings in the literature.

The updated definitions and values for parameters and variables are highlighted in yellow in the revised manuscript for easy identification.

We appreciate your feedback, as it has helped us improve the clarity and accuracy of our presentation.

11 – It is not usual to write “The circumstances for a load” but, for example, “Load cases” or “loading conditions,” which was used in the text.

Response: Thank you for your comment and suggestion regarding the terminology used in our paper. We appreciate your feedback on improving clarity and consistency.

We acknowledge that "loading conditions" is more commonly used than "The circumstances for a load." We have revised the manuscript to replace instances of "The circumstances for a load" with "loading conditions," as appropriate, throughout the text. These changes ensure that our terminology aligns with standard practices in the field.

The revisions have been implemented and highlighted in yellow in the updated manuscript for easy identification.

12 – “Performance Comparison: Among all the average HV values of the algorithms considered, MOHO exhibits the highest values for truss structures, with respective averages of 53518.79, 1894.07, 4087.08, 2317.36, and 75288799”. In this sentence in the second bullet of Section 5, it is necessary to emphasize which experiments obtained these values, although these results are apparent in the tables.

Response:

Thank you for your feedback. Calculating the hypervolume (HV) in the context of multi-objective optimization involves measuring the volume of the space dominated by a set of solutions relative to a reference point. Hypervolume (HV) values play a significant role in the multi-objective optimization of truss structures. Here are the key influences:

1. Performance Metric: HV values serve as a comprehensive metric for evaluating the performance of optimization algorithms. Higher HV values indicate a better spread and convergence of solutions towards the Pareto front.
2. Solution Quality: Algorithms with higher HV values are more effective at finding high-quality solutions that balance multiple objectives, such as minimizing weight and maximizing structural strength.
3. Comparative Analysis: HV values allow for a quantitative comparison between different optimization algorithms, helping to identify the most efficient ones for specific optimization problems.
4. Decision Making: Higher HV values provide engineers and designers with diverse optimal solutions, facilitating better decision-making based on various trade-offs among competing objectives.
5. Optimization Efficiency: By achieving higher HV values, algorithms demonstrate their robustness and efficiency in navigating complex solution spaces and handling multiple constraints inherent in truss structure optimization.

In summary, HV values are crucial for assessing and improving the effectiveness of multi-objective optimization algorithms applied to truss structures, ensuring optimal and well-balanced design solutions.

Some changes have been implemented and highlighted in yellow in the updated manuscript.

13 – It is suggested that the best values in boldface be highlighted in Tables 9 to 13 to make it easier for the reader to see.

Response: Thank you for your valuable suggestion. We agree that highlighting the best values in boldface will improve the tables' readability and clarity. We have updated Tables 9 to 13 to boldface the best values for each category, making it easier for readers to identify the optimal results.

The revised tables are now included in the updated manuscript, and the changes are highlighted in yellow for easy identification.

We appreciate your feedback, which has helped enhance the presentation of our results.

14 – NSGA II is not correctly written in the figure captions.

Response: Thank you for pointing out this issue. We apologise for the oversight. We have reviewed and corrected all "NSGA II" instances in the figure captions to ensure they are appropriately written. The revised figure captions now accurately reflect the correct notation of NSGA-2.

These changes have been implemented throughout the manuscript, and the corrections are highlighted in yellow for easy identification.

15 – Please change structural deformation to structural deflection. Deformation is different from displacement.

Response: Thank you for your observation. We acknowledge that "structural deflection" is a more accurate term in the context of our work, as it specifically refers to displacement. To ensure precise terminology, we have replaced all instances of "structural deformation" with "structural deflection" throughout the manuscript.

The revised text has been updated accordingly, and these changes are highlighted in yellow in the revised manuscript for easy identification.

16 - In the labels of several figures, p10bar was used, etc. It could be 10-bar.

Response: Thank you for your suggestion. While doing an experiment, we used p10bar as the objective function name for a 10-bar truss structure, similarly p25bar for a 25-bar truss, p60bar for a 60-bar truss, p72bar for a 72-bar truss, and p942bar for a 942-bar truss structure. So please consider it accordingly.

Reviewer #2: The results have essential value; I believe the article should be published after the minor revision.

1 - Sufficient information about the previous study findings is presented for readers to follow the present study rationale and procedures.

The authors make a systematic contribution to the research literature in this area of investigation.

The title is adequate for the content discussed in the manuscript: explanatory, brief, and intense.

The article is novel and original, which covers the journal's scope.

The technology applied, and the performance review of the proposed design is demonstrated efficiently.

If required, the authors are informed to justify with real-time experimental analysis in the result and discussion section.

Response: Thank you for your positive and encouraging feedback on our manuscript. We are pleased you find our work valuable, systematic, novel, and original. We appreciate your acknowledgement of the adequacy of our title, the presentation of previous study findings, and the demonstration of our technology and performance review.

Regarding your suggestion to justify real-time experimental analysis in the result and discussion section, we acknowledge the importance of experimental validation. While our current study primarily focuses on theoretical and simulation-based results, we plan to include real-time experimental analysis in our future work to substantiate our findings further.

We have made the minor revisions suggested and have highlighted these changes in the revised manuscript. We hope the updated version meets your expectations and are grateful for your recommendation to publish our article.

Thank you once again for your valuable insights and support.

2 - This article contains significant information on the current area of research.

The overall structure of the article is well-organized and balanced.

The article was written with the minimum length necessary for all relevant information.

However, future enhancements will be described in the conclusion section.

Response: Thank you for your positive feedback on our manuscript. We are glad to hear that you find our article well-organized and contains significant information relevant to the current research area. We appreciate your acknowledging our efforts to present the information concisely yet comprehensively.

Regarding your suggestion to include future enhancements in the conclusion section, we agree that outlining future directions for research is crucial for advancing the field. In response to your comment, we have revised the conclusion section to discuss potential avenues for future research and enhancements.

These revisions have been incorporated into the manuscript, and the updated section is highlighted in yellow for easy identification.

We appreciate your insightful feedback, which has helped improve the completeness and relevance of our article.
